# Nanorobotic System iTRo for Controllable 1D Micro/nano Material Twisting Test

**DOI:** 10.1038/s41598-017-03228-4

**Published:** 2017-06-08

**Authors:** Haojian Lu, Wanfeng Shang, Xueyong Wei, Zhan Yang, Toshio Fukuda, Yajing Shen

**Affiliations:** 1 Mechanical and Biomedical Engineering Department, City University of Hong Kong, Hong Kong, SAR 999077 China; 2 Mechanical Engineering Department, Xi’an University of Science and technology, Xi’an, 710054 China; 3 Mechanical Engineering Department, Xi’an Jiaotong University, Xi’an, 710049 China; 4 Robotics and Microsystems Center, Soochow University, Suzhou, 215021 China; 5 Institute for Advanced Research, Nagoya University, Nagoya, 464-0814 Japan; 6 School of Mechatronic Engineering, Beijing Institute of Technology, Beijing, 100081 China; 7 CityU Shen Zhen Research Institute, Shen Zhen, 518057 China

## Abstract

*In-situ* micro/nano characterization is an indispensable methodology for material research. However, the precise *in-situ* SEM twisting of 1D material with large range is still challenge for current techniques, mainly due to the testing device’s large size and the misalignment between specimen and the rotation axis. Herein, we propose an *in-situ* twist test robot (iTRo) to address the above challenges and realize the precise *in-situ* SEM twisting test for the first time. Firstly, we developed the iTRo and designed a series of control strategies, including assembly error initialization, triple-image alignment (TIA) method for rotation axis alignment, deformation-based contact detection (DCD) method for sample assembly, and switch control for robots cooperation. After that, we chose three typical 1D material, i.e., magnetic microwire Fe_74_B_13_Si_11_C_2_, glass fiber, and human hair, for twisting test and characterized their properties. The results showed that our approach is able to align the sample to the twisting axis accurately, and it can provide large twisting range, heavy load and high controllability. This work fills the blank of current *in-situ* mechanical characterization methodologies, which is expected to give significant impact in the fundamental nanomaterial research and practical micro/nano characterization.

## Introduction

Since the mid-nineteenth century, mechanical test has been an irreplaceable methodology for material study owing to its advantages in offering intuitionistic result. Benefiting from the micro/nano technologies, the *in-situ* SEM mechanical characterization has also become possible in recent decades. Compared with traditional methods, *in-situ* SEM test is able to provide a complete picture of the evolution of micro/nano specimen as it is loaded and fails owing to its real-time imaging ability at high magnification. At current stage, scientists have successfully realized the *in-situ* indention^1–3^, bending^4^ and tension (compression)^5, 6^ test of micro/nano materials, which greatly promotes our fundamental understanding on the micro/nano world.

Twisting test plays an indispensable role among all the three widely accepted mechanical test approaches in material research, i.e., stretching (compressing), bending and twisting. From the view of fundamental study, the grain boundary sliding and crystal twinning phenomenon of material can be observed much easily under twisting^7, 8^. On the other hand, from the view of application, many products and components are subjected to torsional forces during their operation, making twisting test be a powerful approach for device evaluation. To twist the small sample, a commonly used approach is to upgrade the traditional torsion test instrument in accuracy^9, 10^. However, limited to the inherent drawbacks of the traditional instruments in structure and working mechanism, it is hardly possible to align the sample perfectly into the rotation axis of the rotation table, so that the effective gauge need to be lengthened to reduce the influence of the vertical misalignment^11–13^. It not only affect the measurement accuracy, but also make it nearly impossible to integrate within SEM for *in-situ* twisting test. Recently, a nanotube-based torsional NEMS device is reported for nano twisting test^14–16^, which overcomes the drawback of the traditional instrument in both size and accuracy. However, this system only works for very few specific samples due to its limit rotation range, torsion load, and inefficient operation mode.

The rapid progress of robotics, especially micro/nano robotics, provides new opportunities for the precise and efficient operation at small scale^17–20^. Nowadays, robot-aided *in-situ* SEM characterization has been regarded as one of the most powerful mechanical test methods at small scale owing to its real-time imaging ability at nanometer resolution^21–23^. However, current nanorobotic manipulation systems for SEM are mainly subjected to linear motion. Therefore, although they are capable for the *in-situ* stretching (compressing) and bending test effectively, they cannot meet the requirement of twisting test needing precise rotational operation.

The rotational operation inside SEM raises many serious challenges, mainly including the high manipulation flexibility in narrow space; accurate sample rotational positioning in 3D while only 2D SEM surface image information is available; system cooperation for alignment, assembly and twisting operation at microscopy environment. Herein, in this paper, we develop a nanorobotic manipulation system, named *in-situ* twisting test robot (iTRo), to deal with the above challenges. Firstly, the iTRo with six degree-of-freedoms (DOFs) and two independent manipulation units is developed and integrated within SEM. Then, a series of operation and control strategies are proposed to conduct the assembly error calibration, sample alignment, sample assembly and twisting operation. Lastly, torsion test of magnetic microwire, glass fiber, and human hair is implemented and the experimental results are discussed.

## Results

### Development of iTRo

**Figure 1:**
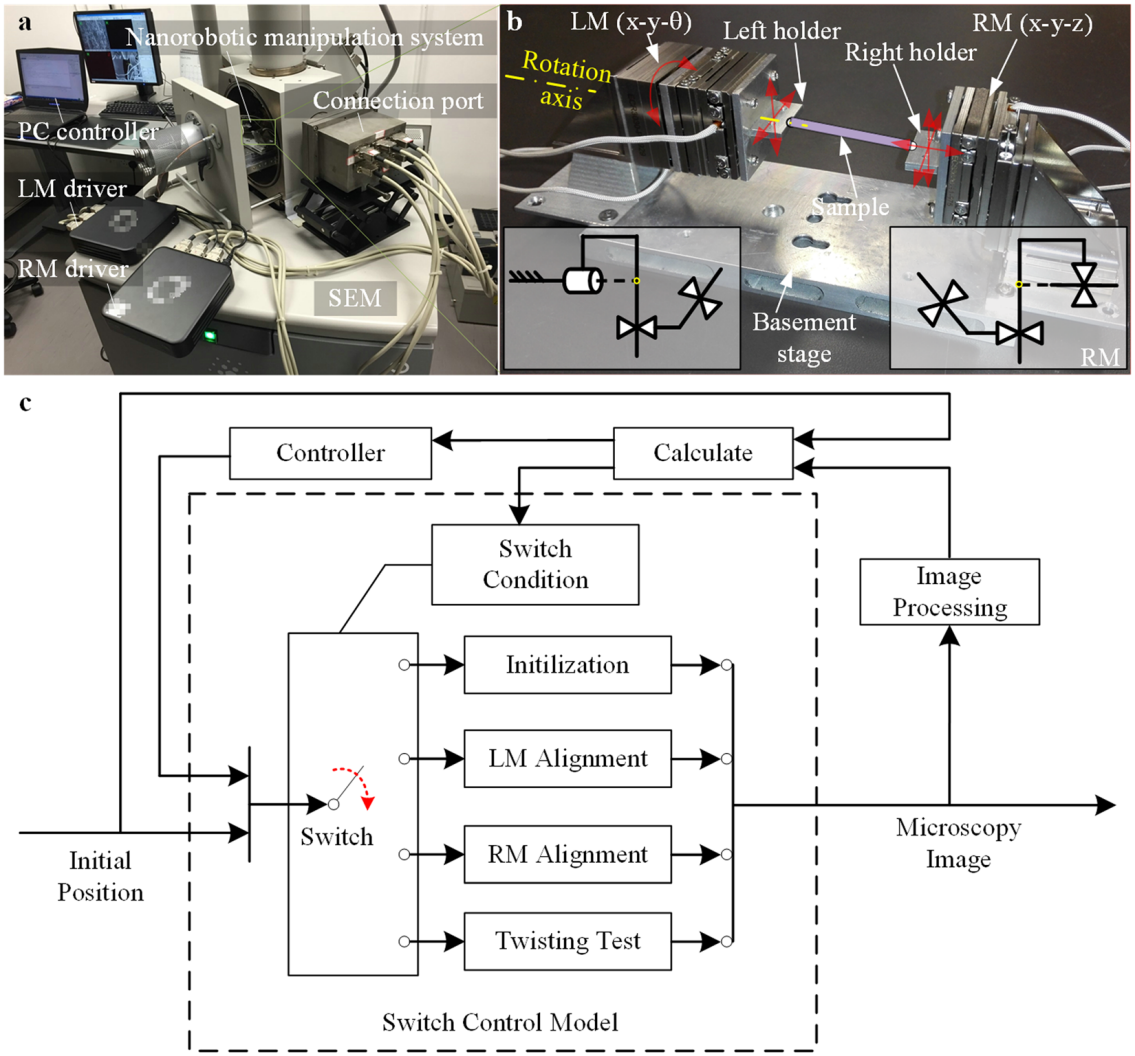
Illustration of the developed iTRo system. (**a**) Overview of system setup. The robot is assembled inside SEM chamber. The PC controller and motor drivers communicate with the robot through a self-designed connection port. (**b**) Image of iTRo. It consists of two independent manipulation units, i.e, LM (x-y-θ) and RM (x-y-z). The two ends of the sample are fixed on the holder of LM and RM respectively. (**c**) Switch control strategy. There are four steps for a fully twisting test process, i.e., initialization, LM alignment, RM alignment and twisting. In each step, SEM image is captured in real time firstly. Then, the position information of the sample and robot is obtained by image processing. After that, proper control signals are send to each nanopositioner to drive the robot.

The iTRo for SEM mainly consists of two independent 3-DOF manipulators, i.e., left manipulator (LM) and right manipulator (RM) on the same basement stage, and a SEM vision feedback system. LM is composed of three independent nanopositioners: two linear positioners and one rotary positioner; and RM is composed by three independent linear positioners. On the end of LM and RM, two sample holders are designed to hold the two ends of sample for twisting test. Additionally, a specific connection port is also designed to control the robot by PC outside SEM’s chamber (Fig. 1a,b and Movie 1).

Here, four steps are designed for a fully *in-situ* twisting test procedure, including robot initialization, LM alignment, RM alignment and twisting operation, which are automated by a switch control strategy (Fig. 1c). In each step, the operation of each nanopositioner is controlled automatically by real-time SEM image processing.

### Robot Initialization

**Figure 2:**
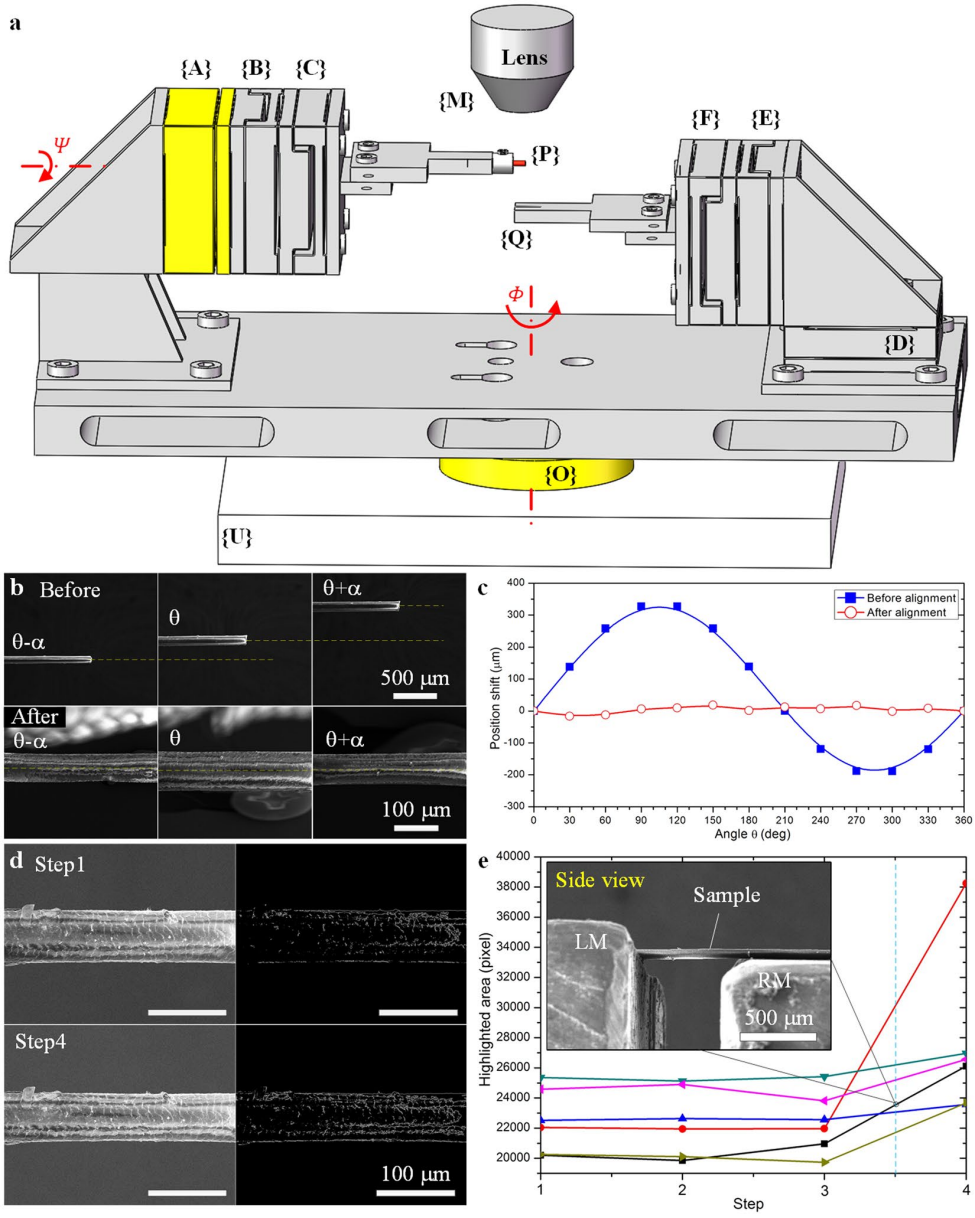
Sample alignment on LM and RM. (**a**) Illustration of the initial assembly errors. *Φ* and *Ψ* are the initial rotational errors on the two rotational joints of the robot. (**b**) Sample’s position before and after LM alignment by TIA approach. α is chosen as 90°. (**c**) Offset Δ*P*_*err*_ in one revolution (360°) before and after LM alignment. (**d**) SEM image and highlight area (unit: pixel) of the sample at initial condition and after deformation, corresponding to the black line in (**e**). (**e**) Changing of the highlighted area during the upward movement of RM. Six experiments are designed independently by taking six different samples. The side view image is taken at step 3.5 corresponding to the sample indicating by black line.

As illustrated in Fig. 2a and Supplementary Fig. 1a, ten coordinates are established for iTRo. The world coordinate {U} is established on the SEM stage and the coordinate {O} is established on the sample rotation stage of SEM. The coordinates {A}, {B}, {C}, {D}, {E} and {F} are established on the six nanopositioners; {P} is established on the endpoint of the sample and {Q} is established on the endpoint of the RM sample holder; {M} is established on the microscopy image system. According to the structure of iTRo, it can be seen that the rotatory assembly errors may come from two joints in coordinates {O} and {A}, which are indicated by *Φ* and *Ψ*, respectively. Because the rotational errors would significantly affect the operation accuracy and even lead to operation failure, they must be calibrated to initialize the robot before twisting test.

In the robot initialization process, the sample’s position in {M} and {P} are obtained from SEM image and robot firstly. After that, the rotation matrix ${R}_{LM\_{\rm{calibration}}}$ between {M} and {P} is calculated based on robot kinematic model (Details are available in methods section). As a result, the initial assembly errors, *Φ* and *Ψ*, are calibrated as *Φ* = 0.0327 rad and *Ψ* = 0.1324 rad finally. After such calibration, we adjust the robot to make *Φ* and *Ψ* infinitely closed to 0 degree. In this case, a standardized rotation matrix ${}_{M}{}^{P}R$ between {P} and {M} corresponding to {U} can be simply represented as a standard matrix:

1 $${}_{M}{}^{P}R=[\begin{array}{ccc}0 & {f}_{y} & 0\\ {f}_{x} & 0 & 0\\ 0 & 0 & -1\end{array}]$$

where *f*_*x*_ and *f*_*y*_ are the digital resolutions of the microscope in the *X*_*M*_- and *Y*_*M*_- axes. Similarly, the rotation matrix between {Q} and {M} corresponding to {U} can also be calibrated and standardized as ${}_{M}{}^{Q}R$.

### Sample alignment on LM

To implement twisting test precisely, the misalignment $${\rm{\Delta }}{P}_{err}={[\begin{array}{ccc}{\rm{\Delta }}{x}_{errP} & {\rm{\Delta }}{y}_{errP} & {\rm{\Delta }}{z}_{errP}\end{array}]}^{{\rm{T}}}$$ between sample’s rotation axis and LM’s rotation axis must be small enough (Supplementary Fig. 1). However, in actual practice, it’s nearly impossible to make Δ*P*_*err*_ be acceptable by manually operation. Herein, we use a triple image alignment (TIA) method to calibrate the offset error and then implement the alignment automatically.

As illustrated in Supplementary Fig. 1b, *P* (*P*_*x*_, *P*_*y*_, *P*_*z*_) is defined as a certain point on the sample’s own rotation axis. Because *Z*_*P*_- axis is parallel to the rotation axis, the value of Δ*z*_*errP*_ would not affect the twisting accuracy. Hence, Δ*z*_*errP*_ is simplified ignored in sample alignment, i.e., to define Δ*z*_*errP*_ = 0. At initial condition, the sample’s position *P*_*o*_ (*P*_*x*_, *P*_*y*_) in {M} is obtained from SEM image firstly. After that, we rotate the sample clockwise by α degrees, and then anticlockwise by 2α degrees. During this process, the coordinate differences along *Y*_*M*_- axis, i.e., Δ*y*_*f*_ and Δ*y*_*b*_ (unit: pixel), are calculated by image processing (Supplementary Fig. 1c,d). After mathematical deduction, the offset of sample to LM’s rotation axis can be represented as (details are available in methods section, Supplementary Table 1):

2 $$\Delta {P}_{err}=[\begin{array}{c}{\rm{\Delta }}{x}_{eerP}\\ {\rm{\Delta }}{y}_{eerP}\\ {\rm{\Delta }}{z}_{eerP}\end{array}]=[\begin{array}{c}\frac{{f}_{y}({\rm{\Delta }}{y}_{f}+{\rm{\Delta }}{y}_{b})}{2(\cos \,\alpha -1)}\\ \frac{{f}_{y}({\rm{\Delta }}{y}_{f}-{\rm{\Delta }}{y}_{b})}{2\,\sin \,\alpha }\\ 0\end{array}],\alpha \in ({0.90}^{^\circ })$$

The rule in equation (2) indicates that the sample can be aligned to LM’s rotation axis theoretically regardless of the initial offset.

In this experiment, we set α = 90°, and *f*_*x*_ = 0.2908, *f*_*y*_ = 0.2885 corresponding to microscopy magnification 500 × . The values of Δ*y*_*f*_ and Δ*y*_*b*_ are directly calculated from the feedback SEM image. By substituting these values into equation (2), we obtain the initial offset $${\rm{\Delta }}{P}_{err}={[\begin{array}{ccc}-69.5 & -257.9 & 0\end{array}]}^{{\rm{T}}}{\rm{\mu }}{\rm{m}}$$, which is then used to adjust LM to eliminate the eccentric error.

As the comparison results shown in Fig. 2b, the sample almost maintains on the same straight line while it’s at angle θ + α, θ, and θ − α. To further evaluate the alignment quality, we define two more parameters, i.e., peak value *e*_*P*_ and standard deviation *e*_*SD*_ as:

3 $${e}_{P}=\,\max |y[i]-\sum _{i=1}^{j}\frac{y[i]}{j}|$$

4 $${e}_{SD}=\sqrt{\frac{1}{j-1}\sum _{i=1}^{j}{(y[i]-\frac{{\sum }_{i=1}^{j}y[i]}{j})}^{2}}$$

where *i* indicates the *i*th images captured during LM’s rotation in 360°, *j* is the total number of captured image, *y*[*i*] is sample’s fluctuation displacement along *y* axis shown in the *i*th image. *e*_*p*_ represents the sample’s largest error from the mean value and *e*_*SD*_ represents the dispersion of sample’s error about *y*- axis while LM rotates in one revolution (360°). During LM alignment experiment, we capture the SEM image every 30°, so that *j* = 12. The results show *e*_*p*_ and *e*_*SD*_ is 258 μm and 197 μm respectively at the initial condition. After TIA process in one iteration, *e*_*p*_ reduces to 22 μm and *e*_*SD*_ reduces to 11 μm, which improves 91.5% and 94.4% respectively (Fig. 2c). As to human hair, the sample’s fluctuation is already much less than sample’s diameter (~50 μm), which accuracy is acceptable for twisting test. Please be noted that the accuracy can be improved further by repeating TIA process for multiple times.

### Sample assembly on RM

After the sample is aligned onto the rotation axis of LM, the next challenge is to assemble the other end of it onto RM. Assume the sample holder of RM is located under the sample at the initial condition (Supplementary Fig. 1b), and the offset error between them is defined as $${\rm{\Delta }}{Q}_{err}={[\begin{array}{ccc}{\rm{\Delta }}{x}_{errQ} & {\rm{\Delta }}{y}_{errQ} & {\rm{\Delta }}{z}_{errQ}\end{array}]}^{{\rm{T}}}$$. SEM could provide clear 2D surface image with nanometer resolution, thereby the relative position of sample and RM stage along *X*_*M*_- and *Y*_*M*_- axis can be seen directly. In actual practice, the displacement error on *X*_*M*_-*Y*_*M*_ plane would not affect the sample assembly accuracy, thereby we ignore Δ*x*_*errQ*_ and Δ*y*_*errQ*_ during RM alignment process, i.e., define ${\rm{\Delta }}{x}_{errQ}={\rm{\Delta }}{y}_{errQ}=0$. Yet, for the distance difference along Z_M_- axis (depth direction), it’s hard to know from SEM image directly and must be calibrated.

To calibrate Δz_*errQ*_, we propose a deformation-based contact detection (DCD) approach. According to the imaging principle of SEM, the electrons on the sample surface would change when sample deforms, which would then cause a brightness change on sample surface. Here, DCD approach is designed based on the above principle: we move RM stage until the highlighted area on sample surface changes, which means RM stage contact and deform the sample. The advantage of DCD approach lies in it only requires a single microscope information to determine the contact state in 3D space.

Here, we first define the highlighted area function *S*_*i*_ of a binaried SEM image,

5 $${S}_{i}={\rm{Area}}\{(x,y)\in {\rm{\Omega }}:f({I}_{i}(x,y))=1\}$$

where *f*(•) is a nonlinear function to exert a binary image processing, *I*_*i*_(*x*, *y*) is a M × N gray image captured from SEM vision system in the *i*th step. The DCD rule is defined as:

6 $${\rm{\Delta }}{z}_{errQ}\approx 0\,(\le 0.5{l}_{step})$$

when

7 $${\rm{\Delta }}S=|{S}_{i+1}-{S}_{0}|\ge \varepsilon \,\,\,\,({S}_{i}\subset {I}_{i}\,(x,y),{S}_{0}\subset {I}_{0}\,(x,y))$$

where *l*_step_ is a constant step size of the *Z*_*M*_- axis linear positioner on RM, which is set as 6.67 μm in this paper, ε is a given judgement criterion, which is defined as 4% of *S*_0_. As the above rule shows, if the highlighted area change Δ*S* satisfies with equation (7), Δ*z*_errQ_ is of a very small value, less than 0.5*l*_step_, and then can be treated as zero. (More details are available in methods section)

As shown in Fig. 2d, corresponding to the black line in Fig. 2e, the total highlighted area is 20210 at the initial condition (step 1), and changes to 26119 (step 4) as RM moves up (along *Z*_*M*_- axis) gradually with *l*_step_. In this condition, equation (7) is satisfied (ΔS = 5909 > 20210 × 4%) and then the step size of the positioner is halved (3.33 μm). Next, RM stage starts to move down for one step, so that the error can be controlled within 3.33 μm, which can be ignored compared with the sample’s size. After such alignment, the end of the sample is fixed on the sample holder of RM by glue (Aron alpha, Toagosei Co. Ltd.) finally. (Data are available in Supplementary Table 2).

### *In-situ* SEM twisting test

Three types of 1D materials, magnetic microwire, glass fiber and human hair are chosen for the *in-situ* torsion test inside SEM, which are on behalf of crystal, amorphous and organic material, respectively.

**Figure 3:**
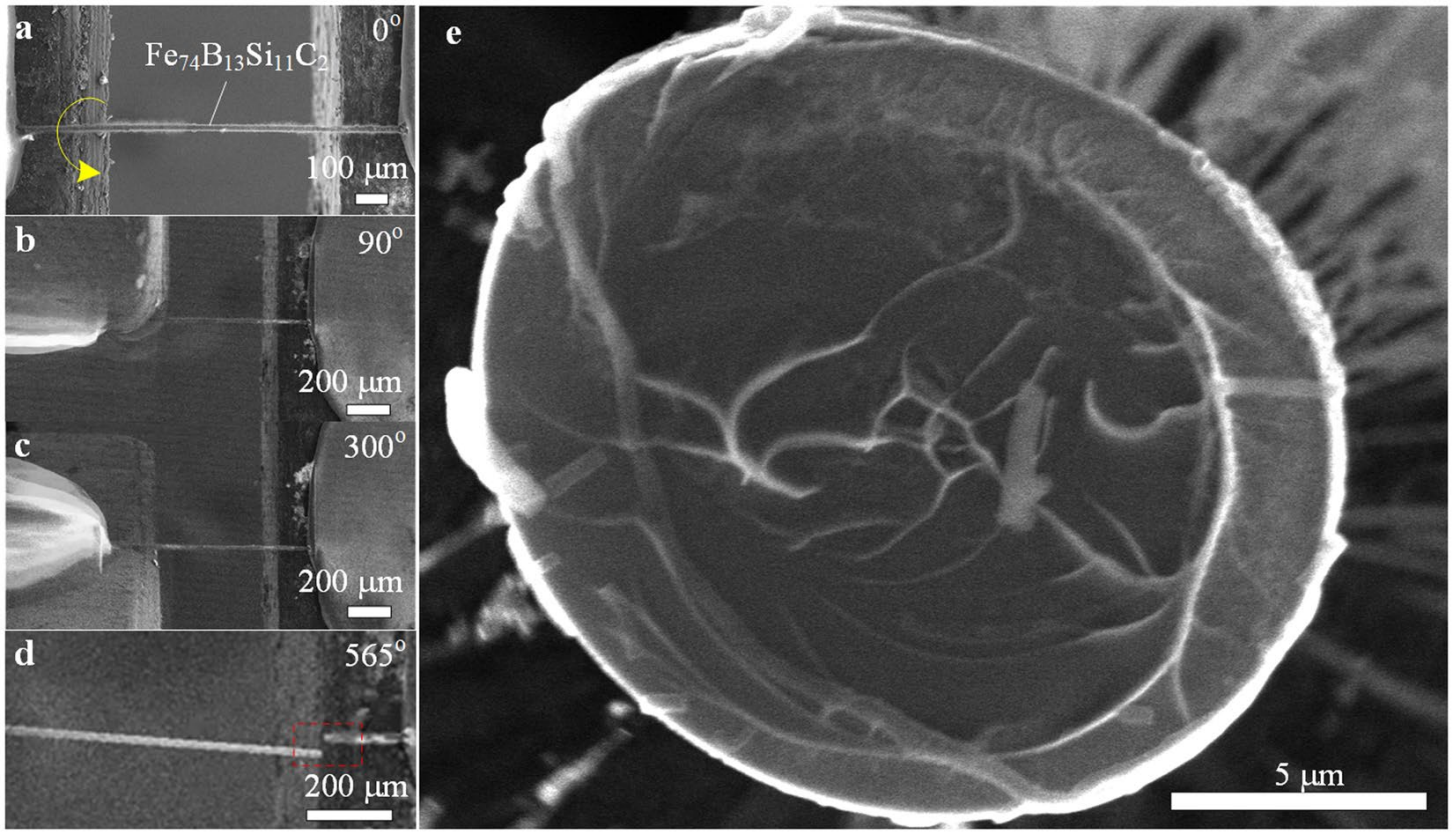
Twisting test of magnetic microwire. (**a**) The microwire is assembled on the two sample holders of iTRo with gauge length approximate 1400 μm. (**b**) and (**c**) gives SEM images at twisting angle 90° and 300° respectively. (**d**) Microwire is broken at twisting angle 565°. (**e**) SEM image of the fracture plane, indicating it’s a typical ductile failure caused by shear stress.

Firstly, a magnetic microwire (Fe_74_B_13_Si_11_C_2_) is aligned and assembled on iTRo following the above processes. Then, we twist the sample in one direction continuously with angular velocity 9.5 deg/s until it’s broken at twisting angle θ = 565° (Fig. 3a,b,c,d). As shown in Fig. 3e, the fracture plane is perpendicular to the rotation axis, clearly indicating it’s a typical ductile failure caused by shear stress. In addition, taking into consideration of the gauge length *L* = 1400 μm and sample radius *R* = 8 μm, we can obtain the shear strain is approximate *R* = θ × *R*/*L* = 5.6%, which is a rational value for ferrous alloy.

**Figure 4:**
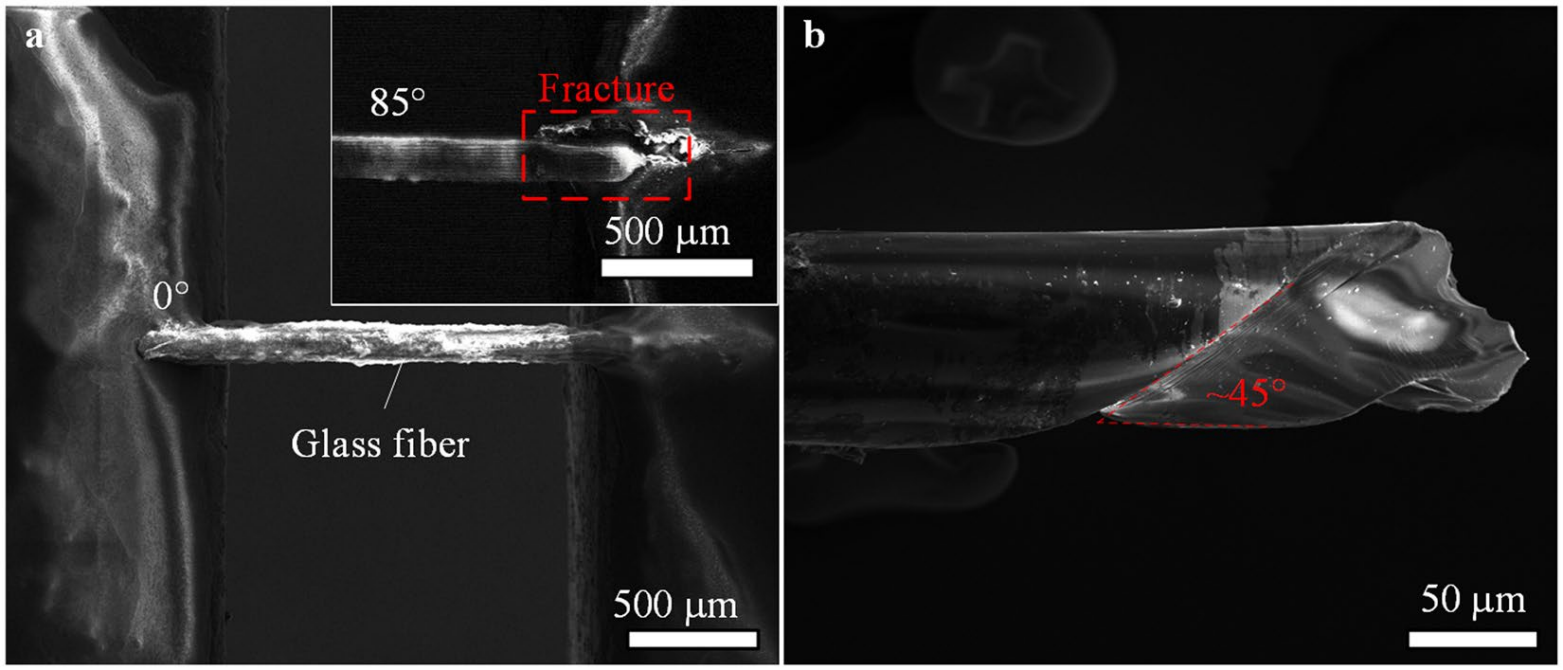
Twisting test of glass fiber. (**a**) The glass fiber is assembled on the two sample holders of iTRo with gauge length approximate 1000 μm, and it is fractured at twisting angle 85°. (**b**) Fracture image indicates that it is a typical brittle failure caused by tensile stress.

Secondly, a single-mode optical fiber (G.652.D) with diameter approximate 100 µm is taken as for twisting test. After the fiber is aligned on iTRo with gauge length 1000 μm, the fiber is twisted with a constant angular velocity 9.5 deg/s until is broken at 85°. It can be seen that the fracture plane is perpendicular to the maximum tensile stress (at 45°) approximately (Fig. 4), i.e., helical fracture, which agrees well with the brittle property of glass.

**Figure 5:**
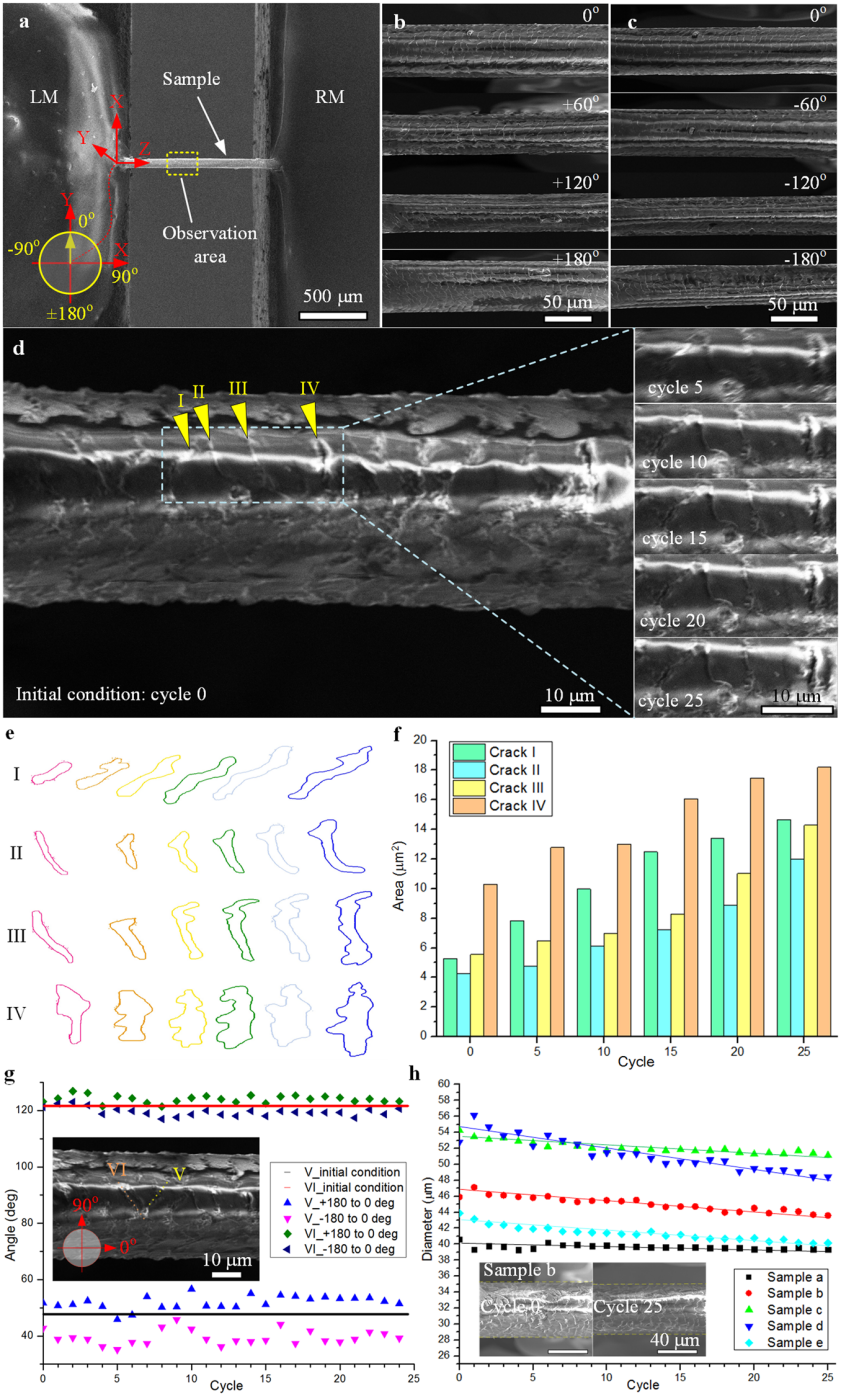
Twisting test of human hair. (**a**) The human hair is assembled on the two sample holders of iTRo with gauge length approximate 680 μm. (**b**) and (**c**) selectively gives four SEM images of the sample while twisting from 0° to +180° and 0° to −180°, respectively. (**d**), (**e**) and (**f**) illustrate the shape change and area increase of the four cracks with increasing of twisting cycle. (**g**) gives the slop difference of the two cracks after the sample is twisting to 0° from +180° and −180° respectively, indicating the slow rebound phenomenal of human hair. (**h**) shows the diameter of human hair (five samples) decreasing gradually with increasing of twisting cycle.

For human hair, we twist it forward-backward for several circles rather than in one individual direction considering its high elasticity. The length between the two ends is approximate 680 μm, as shown in Fig. 5a. The sample is twisted by rotating LM with angular velocity 9.5 deg/s and rotation range ± 180° by 25 circles (Movie 1). Figure 5b,c show images of the sample from 0° to 180° and from 0° to −180° with every 60° in the first twisting circle, clearly demonstrating the torsional deformation on the hair surface (More results are available in Supplementary Fig. 2).

Unlike the traditional materials (crystal and amorphous), as a kind of biomaterial, human air shows more complicate behaviors during twisting. Firstly, we investigate the growth of typical cracks on hair surface. As shown in Fig. 5d, four cracks (I, II, III, IV) on the hair surface are observed by SEM and the images at circle 0th, 5th, 10th, 15th, 20th and 25th are selectively given. Besides, the shape and size change of the cracks with time are also quantitatively given in Fig. 5e,f. The results demonstrate a similar behavior of sample under twisting at macro scale, that the cracks expand gradually with increasing of twisting cycle. For instance, the area of crack IV increased from 10 μm^2^ to 18 μm^2^ after 25 circles, approximate 80% (Data are given in Supplementary Table 3). This phenomenal agrees with the behavior of material under shear stress causing by torsion.

In addition, as shown in Fig. 5g (Data are available in Supplementary Table 4), the slop angel of crack V and VI is 48° and 121° at the initial condition. However, when the hair is twisted from +180° to 0°, the slop angle of crack V and VI increases to 52° and 124° respectively. On contrary, while it is twisted from −180° to 0°, the slop angle of crack V and VI reduces to 39° and 119°, respectively. The above angles are measured in each twisting circle and the results show convinced statistical significance. It indicates the deformation of hair lags the applied extra load, which is an obvious slow rebound effect, which agree with torsional-hysteresis characteristics of human hair in published results^12^.

We also find that the diameter of hair decreases slightly with increase of twisting circles. As shown in Fig. 5h (Data are given in Supplementary Table 5), taken sample b as an example, its diameter reduces from 47 μm to 45 μm after 25 circles. Human hair is a kind of composite biomaterial with complicated multi-layer and porous structure, and has been reported with distinct viscoelasticity^24^. During twisting, the soft components in the hair would be compressed, so to make the diameter become a little smaller. Besides, because of the slow rebound effect of hair, the diameter could not recover to its initial condition immediately. Therefore, the diameter decrease slightly with increasing of twisting circle. This explanation is also verified by a further experiment (Supplementary Fig. 3), that after twisting for 25 circles, the diameter of the hair can recovery (increase ~1.5 μm) after 25 mins standing.

## Discussion

Stretching (or compressing), bending and twisting are three widely accepted mechanical characterization methodologies in material research. However, unlike the other two types of test, *in-situ* SEM twisting test is still very challenge at small scale, since the rotational positioning raise higher demands to the flexibility, accuracy of the assembly, alignment and manipulation. In the proposed iTRo (Fig. 1), we design two independent manipulation units with 6-DOFs in total to ensure the flexibility and employ the nanopositioner to ensure the positioning accuracy. As for SEM, it could only provide the 2D grayscale images. Hence, most of the traditional robotic sensing and positioning techniques are not available, especially for rotational positioning requiring 3D manipulation. Therefore, taking into consideration of the specific features of SEM imaging system, we propose a TIA strategy for sample alignment on LM and a DCD approach for sample alignment on RM (Fig. 2), by which the sample can be assembled precisely. In addition, we also develop a switch control strategy to automate the torsion test process and to guarantee the efficiency (Fig. 1c). As a result, we are able to conduct the *in-situ* torsion test inside SEM effectively (Figs 3, 4 and 5, and Movie 1).

To demonstrate the versatility of iTRo, we test three typical materials: magnetic microwire, glass fiber and human hair. As the results show, the metal microwire’s ductile failure, glass fiber’s helical fracture, and human hair’s crack area extension, diameter change, and slow rebound effect are investigated successfully, which verify the effectiveness and efficiency of iTRo, including the loading ability, twisting range, accuracy and controllability. Specifically, we find some unique behaviors of human hair during the twisting operation. For instance, we can conclude that the cross section of hair is not of a circular shape but more like an irregular ellipse based on the multi-view images (Fig. 5b,c and Supplementary Fig. 2), which is also proofed by the cross-section image of hair (Supplementary Fig. 4). In addition, we also find an obvious slow rebound effect in human hair: 1) the twist deformation of hair lags the twisting angle, which is demonstrated by the slop change of the crack in twisting process (Fig. 5b, Supplementary Table 4); 2) the diameter of hair reduce gradually with increasing of twisting circle (Fig. 5c, Supplementary Table 5), but is able to recovery again after standing for a few minutes (Supplementary Fig. 3). Human hair is a kind of composite biomaterial with complicated multi-layer and porous structure, and has been reported with distinct viscoelasticity^24^. During twisting, the soft components in the hair would be compressed, and the porous structure would also act as damper to slow down the deformation. Thus, the hair shows the slow rebound effect during twisting.

The iTRo provides us the possibility to study the micro/nano material’s behavior under torsion load under SEM *in-situ*, which fills the blanks in the *in-situ* micro/nano material characterization. Except for 1D material, this system could also be used to test 2D material and 3D structures under torsion load. In addition, this system can be easily expended to study the material under composite load, including stretching (compressing), bending and twisting, owing to its high manipulation flexibility and accuracy.

Torque sensing is also a big challenge in micro/nano twisting test^11, 25–27^. However, this paper mainly focuses on solving the operation problems in *in-situ* SEM twisting test, i.e., precise assembly and positioning, especially the misalignment problem in existing twisting test approach^11, 13^. Up to now, we have been able to investigate the sample’s behavior during twisting and study the fracture plane after twisting. As future work, we will cooperate with other researches in sensing field to integrate iTRo with torsion sensors, and then to investigate the micro/nano material’s property more deeply.

## Methods

### Nanorobotic manipulators

LM and RM are assembled on the same basement stage, and they are independent to each other. LM is mainly composed of three nanopositioners: two linear positioners (ECS3030, Attocube Inc.) and one rotary positioner (ECR3030, Attocube Inc.); and RM is composed by three linear positioners (ECS3030, Attocube Inc.). The travel range, resolution, repeatability, maximum drive velocity and maximum load of the linear positioner are 20 mm, 1 nm, 50 nm, 4.5 mm/s and 90 N respectively. The travel range, resolution, repeatability, maximum drive velocity and maximum load of the rotary positioner are 360° endless, 1 μ°, 5% over full range, 10°/s and 20 N respectively.

### Sample preparation

The magnetic microwire consists of two layer initially, the inner metal core (Fe_74_B_13_Si_11_C_2_) and the coated shell (glass). In the twisting experiment, we twist the microwire directly without removing the glass shell. Because the glass is more brittle than metal, so it is teared off before metal broken during twisting. Thus, only the inner metal core is left after fracture. The hair is taken from the experimenter, i.e., the author of this paper. The diameter of the hair is approximate 40–60 μm regarding to various sample. In all experiments, the hair is tested without any chemical or physical treatment. In the cross section observation (Supplementary Fig. 4), the hair is cut by a scissor.

### Center line recognition

Considering the nature of twisting test, the center line of the sample should be taken as the reference during the experiment. Taken a human hair (diameter ~50 μm) as example, its image is captured from the microscopy firstly (Supplementary Fig. 5a). Then, Gaussian smoothing filter is adopted to reduce the noise. After that, the edge of sample is recognized by Canny detection, as the red dots shown in Fig. S5B. To find out the center line, the middle point of the sample *y*_center_ is calculated firstly by:

8 $${y}_{center}=\frac{k\cdot TP+\sum _{m=1}^{k}{y}_{m}-\sum _{n=1}^{k}{y}_{n}}{2k}$$

where *TP* is the height of the full screen, *y*_*m*_ is the distance between sample’s upper edge and image’s upper edge, *y*_*n*_ is the distance between sample’s bottom edge and image’s bottom edge, respectively. In addition, *k* is the number of pixels extracted from the top/bottom edge of the sample, which is defined as *k* = 30 in our experiment.

After the middle point is calculated, a straight line is drawn to indicate the center line of the sample, as the yellow line shown in Supplementary Fig. 5b. This approach is able to ensure the recognition accuracy as high as to two pixels, which is adequate for the automatic robot manipulation.

In material study, the sample for twisting test is usually prepared to a symmetrical structure. Hence, the above center line recognition process can work for most cases in actual practice. Moreover, note that the application scope of TIA approach is not limited to symmetrical sample, since it’s based on a to-be-aligned point. For the unsymmetrical sample, the reference point on twisting axis can be pointed out by operator first. After that, the centerline can be set following the same method mentioned above.

### Details for calibrating the initial assembly error

Based on the robot kinematic model, the rotation matrix between {P} and {M} corresponding to {U} can be represented as:

9 $${R}_{LM\_\mathrm{calibration}}=[\begin{array}{ccc}-{f}_{x}\,\sin \,\varphi  & {f}_{y}\,\cos \,\psi \,\cos \,\varphi  & -\cos \,\varphi \,\sin \,\psi \\ {f}_{x}\,\cos \,\varphi  & {f}_{y}\,\cos \,\psi \,\sin \,\varphi  & -\sin \,\varphi \,\sin \,\psi \\ 0 & -{f}_{y}\,\sin \,\psi  & -\cos \,\psi \end{array}]$$

where *f*_*x*_ and *f*_*y*_ are the digital resolutions of the microscope in the *X*_*M*_- and *Y*_*M*_- axes.

Define we have *n* image positions $${S}_{Mi}={[\begin{array}{ccc}{x}_{Mi} & {y}_{Mi} & {z}_{Mi}\end{array}]}^{{\rm{T}}}$$ of the sample measured from the microscopy image and the corresponding sample positions $${S}_{Pi}={[\begin{array}{ccc}{x}_{Pi} & {y}_{Pi} & {z}_{Pi}\end{array}]}^{{\rm{T}}}$$ obtained from the robot movement, i = 1,2, …*n*. Then, the initial rotatory errors *Φ* and *Ψ* can be calculated by solving the following matrix,

10 $$[\begin{array}{cccc}{S}_{P1} & {S}_{P2} & \mathrm{...} & {S}_{Pn}\end{array}]=[\begin{array}{cccc}{R}_{LM\_\mathrm{calibration}}\cdot {S}_{M1} & {R}_{LM\_\mathrm{calibration}}\cdot {S}_{M2} & \mathrm{...} & {R}_{LM\_\mathrm{calibration}}\cdot {S}_{Mn}\end{array}]$$

Through calculating the inverse matrix of $$[\begin{array}{cccc}{S}_{P1} & {S}_{P2} & \mathrm{...} & {S}_{Pn}\end{array}]$$ by the Moore-Penrose matrix inverse method, the rotation matrix ${R}_{LM\_\mathrm{calibration}}$ can be obtained by the following equation:

11 $${R}_{LM\_\mathrm{calibration}}=[\begin{array}{cccc}{S}_{P1} & {S}_{P2} & \mathrm{...} & {S}_{Pn}\end{array}]\times {({[\begin{array}{cccc}{S}_{M1} & {S}_{M2} & \mathrm{...} & {S}_{Mn}\end{array}]}^{{\rm{T}}}\times [\begin{array}{cccc}{S}_{M1} & {S}_{M2} & \mathrm{...} & {S}_{Mn}\end{array}])}^{\mbox{'}}\times {[\begin{array}{cccc}{S}_{M1} & {S}_{M2} & \mathrm{...} & {S}_{Mn}\end{array}]}^{{\rm{T}}}$$

Note that the value of n depends on the required calibration accuracy of the system, the larger the value, the greater the precision. In this manuscript, we chose *n* = 6 under the magnification of 500×, and then ${R}_{LM\_{\rm{c}}{\rm{a}}{\rm{l}}{\rm{i}}{\rm{b}}{\rm{r}}{\rm{a}}{\rm{t}}{\rm{i}}{\rm{o}}{\rm{n}}}$ is calculated as:

12 $${R}_{LM\_{\rm{c}}{\rm{a}}{\rm{l}}{\rm{i}}{\rm{b}}{\rm{r}}{\rm{a}}{\rm{t}}{\rm{i}}{\rm{o}}{\rm{n}}}=[\begin{array}{ccc}-0.0095 & 0.2858 & -0.1320\\ 0.2907 & 0.0094 & 0.0043\\ 0 & -0.0381 & -0.9912\end{array}]$$

As a result, the initial assembly error *Φ* and *Ψ* are calibrated as *Φ* = 0.0327 rad and *Ψ* = 0.1324 rad, respectively.

### Process of the TIA approach for LM alignment

As illustrated in Supplementary Fig. 1, at the initial condition, define the position of the sample in the rotation axis coordinate {$\widehat{O}$} is $${P}_{0}={[\begin{array}{ccc}{x}_{{P}_{0}} & {y}_{{P}_{0}} & {z}_{{P}_{0}}\end{array}]}^{{\rm{T}}}$$, and in the microscopy coordinate {M} is $${P}_{0\_M}={[\begin{array}{ccc}{x}_{0} & {y}_{0} & {z}_{0}\end{array}]}^{{\rm{T}}}$$. The relationship between *P*_0_ and *P*_0*_M*_ can be represented as:

13 $${P}_{0}={}_{M}{}^{P}R\cdot {P}_{0\_M}$$

After that, if the sample is rotated clockwise by α degrees, we will have two new position of the sample, i.e., $${P}_{1}={[\begin{array}{ccc}{x}_{{P}_{1}} & {y}_{{P}_{1}} & {z}_{{P}_{1}}\end{array}]}^{{\rm{T}}}$$ in {$\widehat{O}$} and $${P}_{1\_M}={[\begin{array}{ccc}{x}_{1} & {y}_{1} & {z}_{1}\end{array}]}^{{\rm{T}}}$$ in {M}. The relationship between *P*_1_ and *P*_1*_M*_ can be represented as:

14 $${P}_{1}={}_{M}{}^{P}R\cdot {P}_{1\_M}$$

Similarly, after the sample is rotated anticlockwise by 2α degrees, the relationship between *P*_2_ and *P*_2*_M*_ can be represented as:

15 $${P}_{2}={}_{M}{}^{P}R\cdot {P}_{2\_M}$$

Based on the robotic kinetic mode, the relationship between *P*_0_ and *P*_1_, *P*_0_ and *P*_2_ can be represented as:

16 $${P}_{1}=R(\alpha )\cdot {P}_{0}$$

17 $${P}_{2}=R(-2\alpha )\cdot {P}_{1}$$

where *R*(*α*) is the rotation matrix:

18 $$R(\alpha )=[\begin{array}{ccc}\cos \,\alpha  & \sin \,\alpha  & 0\\ -\sin \,\alpha  & \cos \,\alpha  & 0\\ 0 & 0 & 1\end{array}]$$

As illustrated in the microscopy image (Supplementary Fig. 1), we get:

19 $$[\begin{array}{c}{\rm{\Delta }}{x}_{f}\\ {\rm{\Delta }}{y}_{f}\\ {\rm{\Delta }}{z}_{f}\end{array}]={P}_{1\_M}-{P}_{0\_M}$$

and

20 $$[\begin{array}{c}{\rm{\Delta }}{x}_{b}\\ {\rm{\Delta }}{y}_{b}\\ {\rm{\Delta }}{z}_{b}\end{array}]={P}_{2\_M}-{P}_{0\_M}$$

where the coordinate difference along *Y*_*M*_- axis, i.e., Δ*y*_*f*_ and Δ*y*_*b*_, can be calculated based on image processing. During the rotational movement, Δ*z*_*f*_ and Δ*x*_*b*_ equal to zero. By solving the above equations (13)–(20), the initial eccentric position *P*_*0*_, or say Δ*P*_*err*_ can be obtained as equation (2):

$${\rm{\Delta }}{P}_{err}={P}_{0}=[\begin{array}{c}{x}_{{P}_{0}}\\ {y}_{{P}_{0}}\\ {z}_{{P}_{0}}\end{array}]=[\begin{array}{c}\frac{{f}_{y}({\rm{\Delta }}{y}_{f}+{\rm{\Delta }}{y}_{b})}{2\,(\cos \,\alpha -1)}\\ \frac{{f}_{y}({\rm{\Delta }}{y}_{f}-{\rm{\Delta }}{y}_{b})}{2\,\sin \,\alpha }\\ 0\end{array}]$$

Based on the above process, the offset Δ*P*_*err*_ of the sample from the rotation axis of LM can be calculated easily by rotating the robot for α and −α from the initial condition^28^. Details for the alignment process are illustrated in Supplementary Table 1.

### Process of the DCD approach for RM alignment

The process of DCD approach for RM alignment is illustrated in Supplementary Table 2. During the experiment, the robot moves upward with a constant step size and the highlighted area change Δ*S* is calculated dynamically in each step. Once the value of Δ*S* is larger than the criterion ε, which means the robot touches and deforms the sample, the robot would move backward for half-step and then stop. In this paper, the step size is chosen as 6.67 μm. Note that if a smaller step size is chosen, the sample alignment accuracy of Δ*z*_*errQ*_ can be improved further.

The calculation of highlighted area *S*_*i*_ is conducted by image processing. Firstly, the captured grey-scale SEM image (1024 × 800) is converted to binary image. In this paper, we adopt quasi-auto threshold for every sample and then a threshold value is achieved by the image process system. To judge the highlighted area by the same standard, the threshold must be served as a fixed value in the following alignment process. After binaryzation, the highlighted area in SEM image is recognized by contour extraction using Suzuki algorithm^29^. The algorithm chooses external contour of each highlighted area and store nearly all the points in vector. The distance between two close contour points $C{P}_{1}\,({{\rm{x}}}_{CP\_1},{{\rm{y}}}_{CP\_1})$ and $C{P}_{2}\,({{\rm{x}}}_{CP\_2},{{\rm{y}}}_{CP\_2})$ should not exceed 1 pixel, which means:

21 $${\max (\mathrm{abs}({\rm{x}}}_{CP\_1}-{{\rm{x}}}_{CP\_2}),{\mathrm{abs}({\rm{y}}}_{CP\_1}-{{\rm{y}}}_{CP\_2}))=1$$

At last the algorithm will calculate the close highlighted area by Green’s theorem:

22 $$G={\oint }_{H}x\cdot dy=-{\oint }_{H}y\cdot dx=\frac{1}{2}{\oint }_{H}(-y\cdot dx+x\cdot dy)$$

where *H* is a positively oriented, piecewise smooth, simple closed curve in a plane, and let *G* be the region bounded by *H*.

Lastly, the highlighted area *S*_*i*_ is calculated by counting all the circled pixels. For the sample of human hair, the value *ε* is defined as 4% of the initial highlighted area *S*_*0*_ (unit: pixel). Note that for other types of samples with totally different imaging properties, the value of *ε* may need slight adjustment experimentally to achieve higher accuracy. In this paper, we didn’t chose the amount of the highlighted pixels in a binary image, but added one more step to find the contours in the binary image and took the circled areas as the highlighted area. Compared with accounting highlight pixels in a binary image, the contour border extraction method of highlighted area could eliminate the random noise and detect the deformation more exactly (Supplementary Fig. 6).

### Data and materials availability

All data needed to evaluate the conclusions in the paper are present in the paper and/or the Supplementary Materials. Additional data related to this paper may be requested from Y. Shen (yajishen@cityu.edu.hk).

## Electronic supplementary material

Supporting material

Twisting test video
