# Supplementary material for: Nanorobotic System iTRo for Controllable 1D Micro/nano Material Twisting Test
Source: Sci Rep. 2017 Jun 8;7:3077. doi: 10.1038/s41598-017-03228-4 (PMC5465204; doi:10.1038/s41598-017-03228-4)
Supplement: Supplementary file 1 — Supporting material [file 41598_2017_3228_MOESM1_ESM.pdf]

Supplementary Materials

Nanorobotic System iTRo for Controllable 1D Micro/nano Material Twisting Test

Haojian Lu,[1] Wanfeng Shang,[2] Xueyong Wei[3], Zhan Yang[4], Toshio Fukuda,[5,6] and Yajing Shen[1,7,*]

1. Mechanical and Biomedical Engineering Department, City University of Hong Kong, Hong Kong SAR 999077, China
2. Mechanical Engineering Department, Xi'an University of Science and technology, Xi'an 710054, China
3. Mechanical Engineering Department, Xi'an Jiaotong University, Xi'an 710049, China
4. Robotics and Microsystems Center, Soochow University, Suzhou 215021, China
5. Institute for Advanced Research, Nagoya University, Nagoya 464-0814, Japan.
6. School of Mechatronic Engineering, Beijing Institute of Technology, Beijing 100081, China
7. CityU Shen Zhen Research Institute, Shen Zhen 518057, China

correspondence to: yajishen@cityu.edu.hk

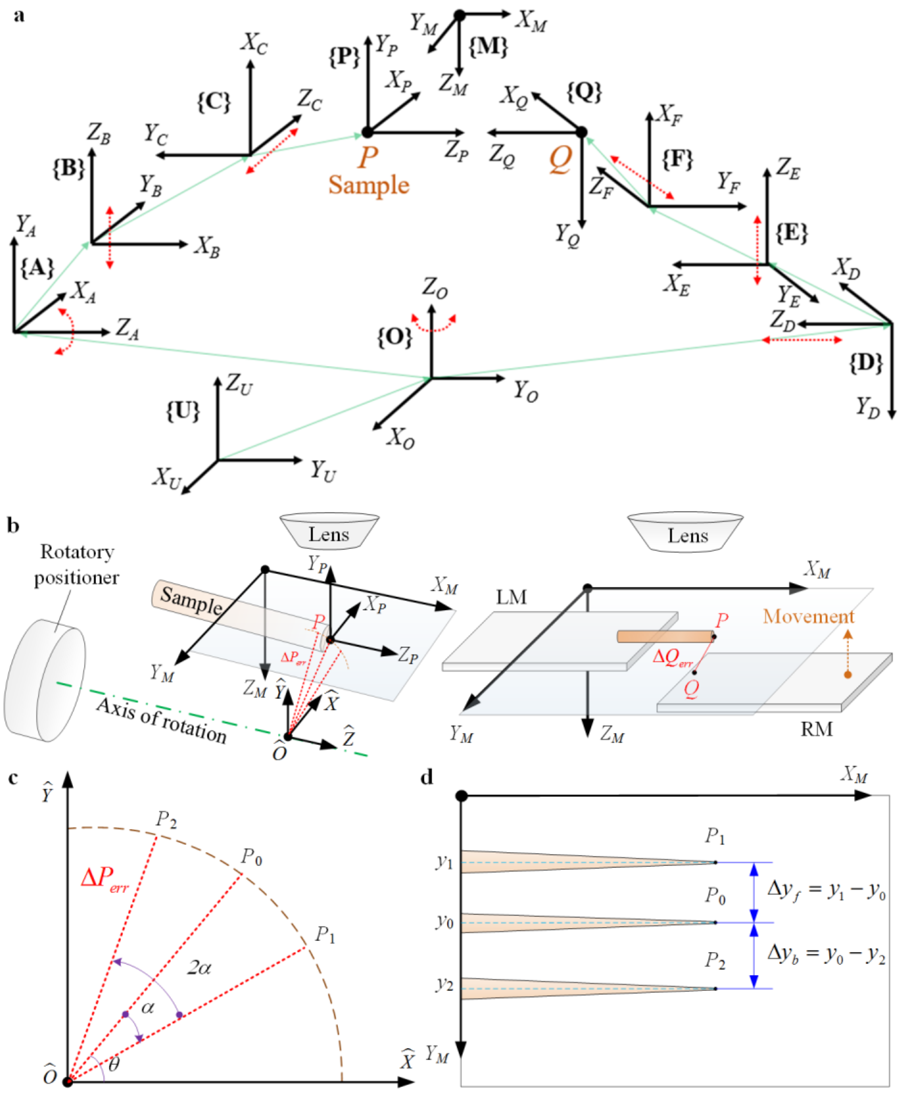

Supplementary Fig. 1. Robot coordinate system and kinematic model. (**a**) Ten coordinate systems of iTRo. The world coordinate {U} is established on the SEM stage and the coordinate {O} is established on the sample rotation stage of SEM. The coordinates {A}, {B}, {C}, {D}, {E} and {F} are established on the six nanopositioners; {P} is established on the endpoint of the sample and {Q} is established on the endpoint of the RM sample stage; {M} is established on the microscopy image system. The point P and Q indicates the endpoint of the sample and the RM sample stage. (**b**) Illustration of the relative position of sample, LM and RM. (**c**) and (**d**) show the three positions of sample and their differences in TIA alignment process.

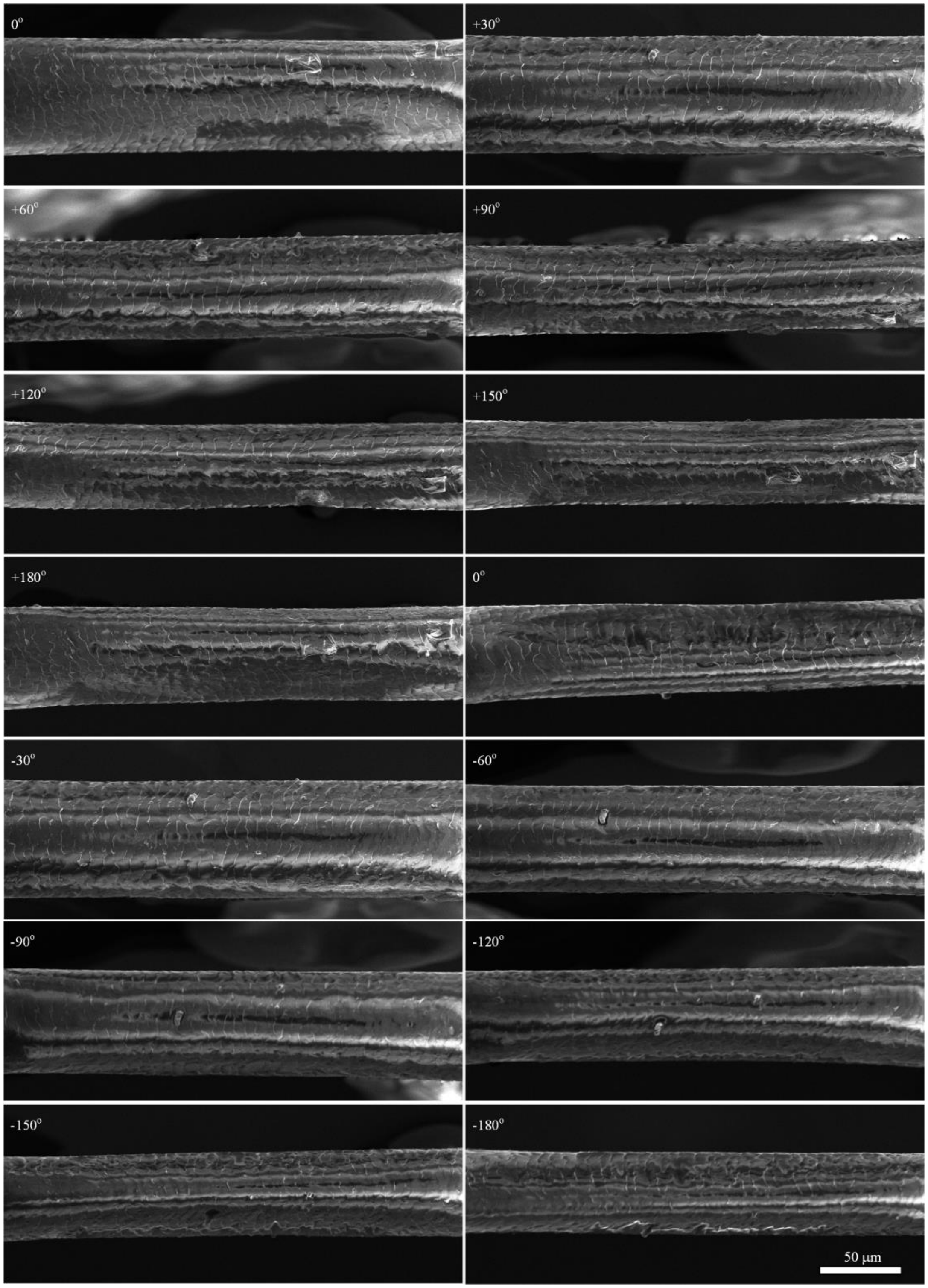

Supplementary Fig. 2. Images of human hair every 30° in the first twisting circle, which indicate: 1) Human hair can be twisting with a large twisting angle without fraction. 2) The diameter of the human is different while observing it from different direction. 3) The change of texture direction on the surface can be observed.

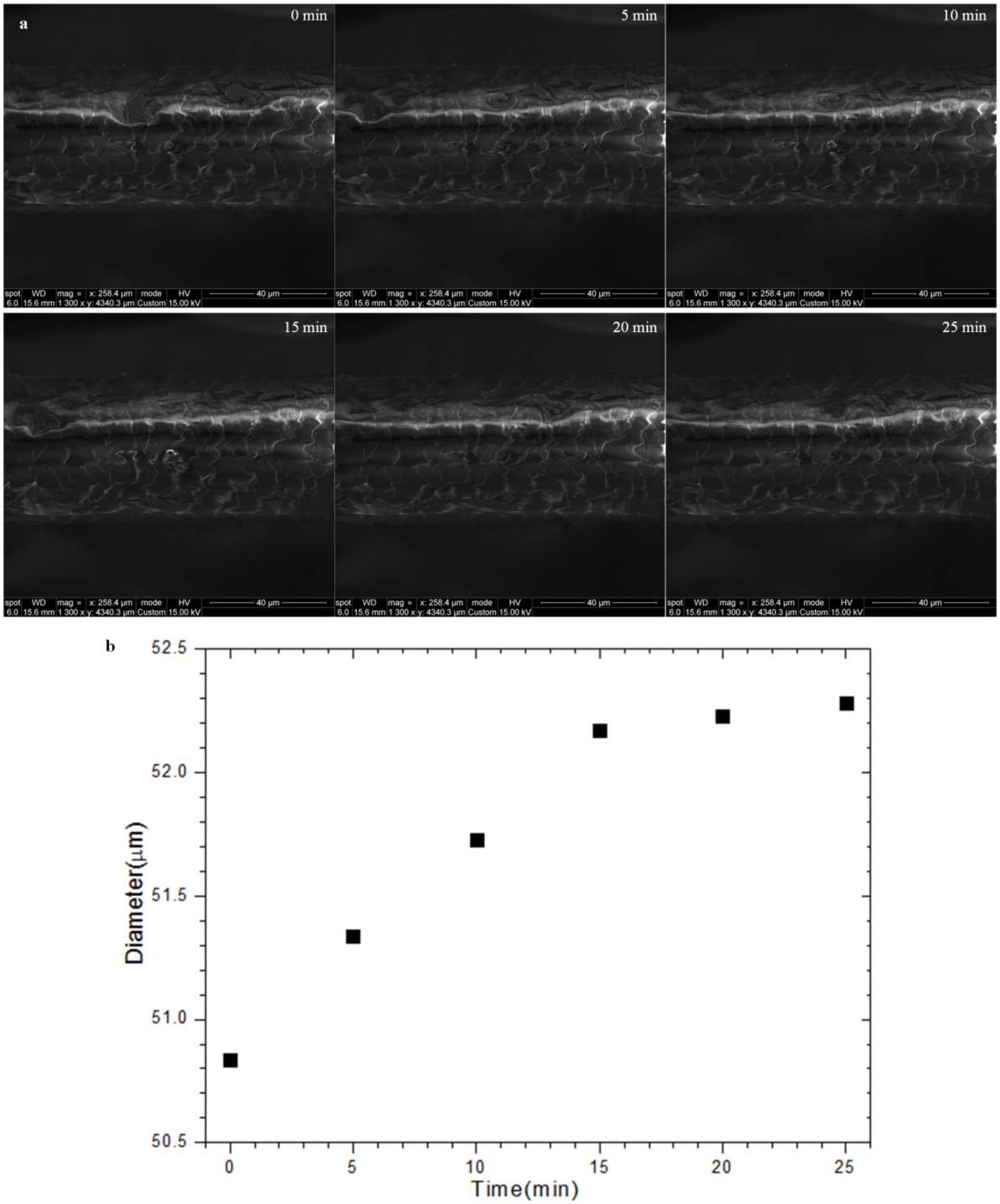

Supplementary Fig. 3. Diameter recovery of hair after standing for 25 mins. (**a**) After twisting test for 25 circle, the robot moves back to its initial condition (twisting angle=0°). Then, the hair is kept still for 25 mins. The image of hair is taken every 5 mins. (**b**) Diameter change v.s. time. The diameter gradually increase, indicating the hair is recovering from twisting deformation after unload.

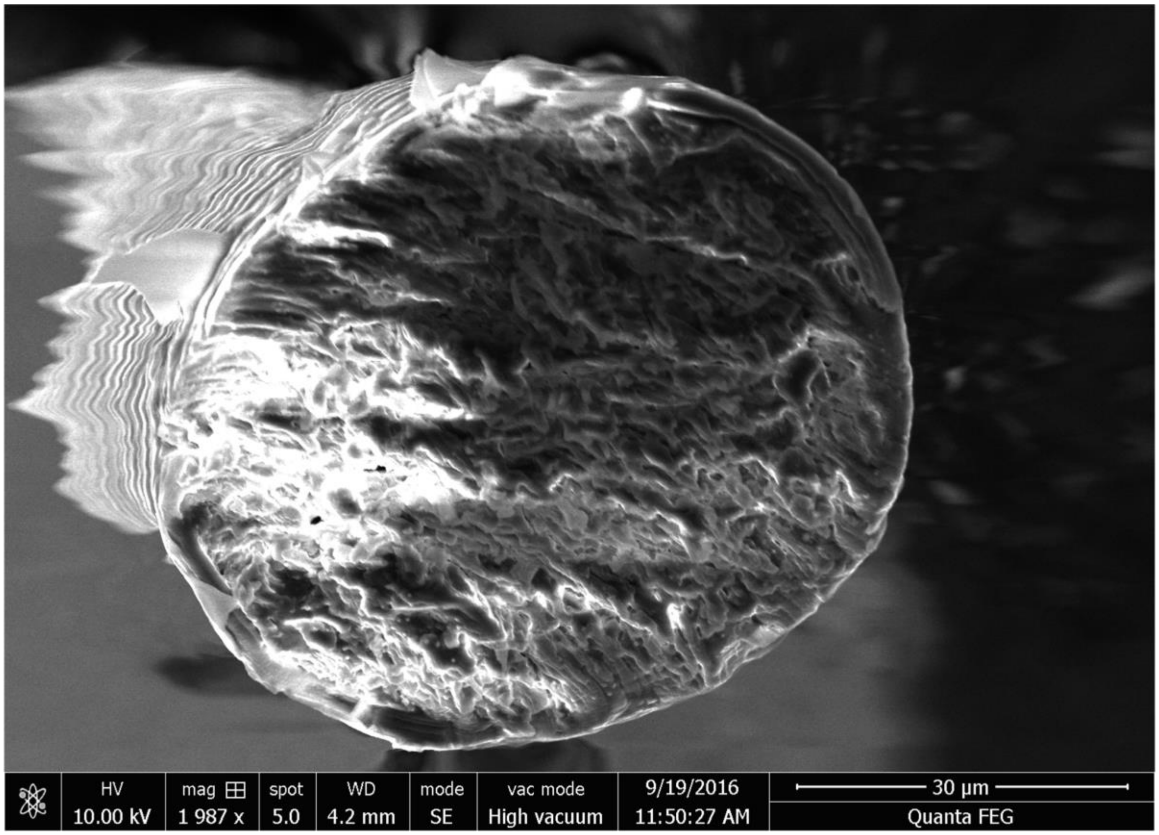

Supplementary Fig. 4. Cross section image of the human hair, indicating the cross section is not ideal circular shape. The hair is cut by a scissor and then observed by SEM without any chemical treatment.

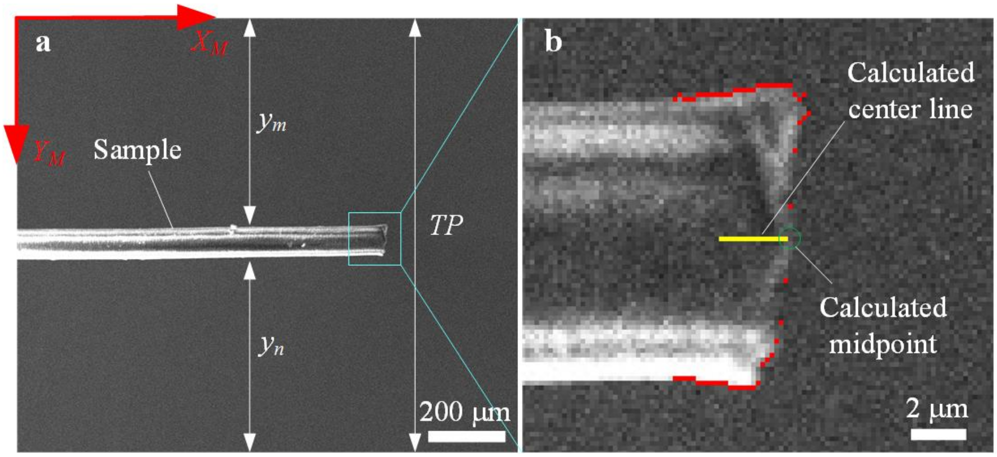

Supplementary Fig. 5. Center line recognition. (**a**) Illustration for the centerline recognition. (**b**) Enlarge image of the tip. The red dots indicate the recognized points. The green circle and yellow line indicates the calculated midpoint and center line, respectively.

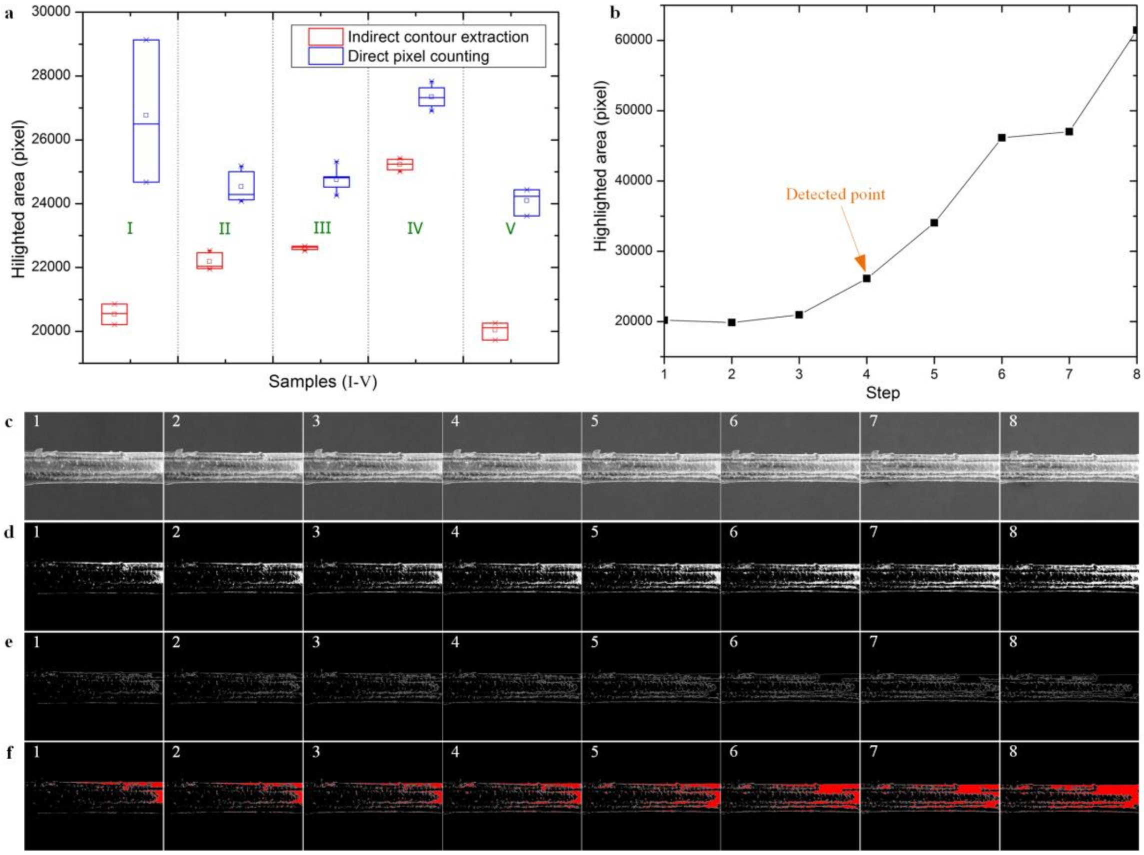

Supplementary Fig. 6. Image processing in DCD method. (**a**) Comparison of the direct pixel counting method and the indirect contour extraction method. The later one used in this paper is more stable. (**b**) Highlight area change in each step, corresponding to the black line in Fig. 3. (**c**) Initial SEM gray-scale image. (**d**) Binary images. (**e**) Highlighted area by contour extraction. (**f**) Highlighted area filled by red color.

Supplementary Table 1.

Algorithm for sample alignment on LM.

**Algorithm 1** TIA algorithm for sample alignment onto the rotation axis
Step 1: Calculate the initial sample position P_0 in microscopy coordinate {M}.
Step 2: Rotate the sample by α degree, and calculate the sample position P_1 in {M}.
Step 3: Rotate the sample by -2α degree, and calculate the sample position P_2 in {M}.
Step 4: Calculate the offset of the sample ΔP_{err} based on equation (2).
Step 5: Move the robot to align the sample to the rotation center.
Step 6: Repeat step 1-3, calculate the maximum difference e among P_0, P_1, P_2.
Step 7: If e is acceptable (in this manuscript, the stopping criterion e is chosen as a quarter of the sample's diameter), stop the alignment; otherwise, repeat step 1-6.

Supplementary Table 2.

Algorithm for sample alignment on RM.

**Algorithm 2** DCD algorithm for sample alignment on RM
Setp 1: Calculate highlighted area of the initial image: S_0.
Step 2: Initialize $\varepsilon = S_0 \times 4\%$, a fixed motor step size Δd.
Step 3: RM moves up along Z_M- axis by Δd.
Step 4: Calculate highlighted area of the current image in step *i*: S_i.
Step 5: Compute $\Delta S = \lvert S_i - S_0 \rvert$.
Step 6: If $\Delta S \geq \varepsilon$ then goes to Step 7, else goes to Step 3.
Step 7: Halve the step size $\Delta d = \Delta d / 2$.
Step 8: RM moves down for one step.

Supplementary Table 3.

Crack area change (μm^2) during twisting test.

Cycle	Crack I	Crack II	Crack III	Crack IV
0	5.3	4.3	5.6	10.3
5	7.8	4.8	6.5	12.8
10	10.0	6.1	7.0	13.0
15	12.5	7.2	8.3	16.1
20	13.4	8.9	11.0	17.5
25	14.7	12.0	14.3	18.2

Supplementary Table 4.

Slop angle (degree) change of crack V and VI during twisting test.

Cycle	Crack V		Crack VI	
	$+180^{o}$ to 0°	-180° to 0°	$+180^{o}$ to 0°	-180° to 0°
0	48.1	48.1	121.3	121.3
1	51.7	42.8	123.2	121.2
2	50.8	38.8	124.3	122.5
3	51.2	39.4	126.9	123.0
4	52.5	38.8	126.3	121.9
5	50.5	36.2	121.5	118.8
6	45.9	35.2	125.1	120.4
7	47.5	37.8	124.4	119.9
8	54.0	37.4	122.7	119.0
9	50.2	43.2	121.4	117.0
10	50.3	45.8	123.3	117.6
11	56.6	42.4	124.5	118.6
12	50.7	38.9	125.1	120.0
13	50.5	36.2	124.1	118.5
14	50.3	38.3	123.0	118.1
15	55.1	38.0	125.4	119.9
16	51.2	38.5	122.6	118.1
17	54.5	44.1	125.0	119.9
18	54.1	37.4	125.4	119.4
19	53.1	41.8	124.1	119.3
20	53.9	38.3	125.0	119.3
21	53.3	37.9	124.6	119.3
22	53.4	38.9	123.3	117.4
23	53.6	41.4	124.1	120.4
24	52.3	40.9	123.3	118.7
25	51.5	39.2	123.2	120.7

Supplementary Table 5.

Diameter change (μm) of human hair during twisting test.

Cycle	Sample a	Sample b	Sample c	Sample d	Sample e
0	40.6	45.9	54.3	52.8	43.9
1	39.3	47.1	53.4	56.1	43.1
2	39.8	46.2	53.1	54.7	42.5
3	39.7	46.1	53.3	53.6	42.4
4	39.3	45.9	52.9	54.0	42.0
5	39.5	45.8	52.7	52.3	41.9
6	40.2	46.0	52.2	53.6	42.1
7	39.9	45.5	52.8	53.0	41.6
8	40.0	45.3	52.3	52.5	41.6
9	39.8	45.4	52.1	51.0	41.5
10	39.8	45.5	51.7	51.5	41.4
11	39.7	45.4	52.0	51.2	41.3
12	39.7	45.5	51.7	51.3	41.2
13	39.6	45.0	51.8	50.7	41.6
14	39.6	44.7	51.7	50.1	41.1
15	39.6	44.7	51.8	50.3	41.2
16	39.6	44.7	51.7	50.2	40.8
17	39.5	44.4	51.7	50.6	40.7
18	39.5	44.0	51.9	50.4	40.7
19	39.5	44.0	51.5	49.1	40.6
20	39.4	44.5	51.3	49.5	40.8
21	39.3	43.9	51.4	49.3	40.3
22	39.4	44.4	51.3	49.3	40.6
23	39.6	44.1	51.7	49.1	40.2
24	39.4	43.7	51.4	48.4	40.1
25	39.3	43.6	51.1	48.4	40.1

Supplementary Movie 1
In-situ SEM twisting test by iTRo system.
